# ANFIS algorithm for mapping computational data of water reservoir homogenization with air bubble flows

**DOI:** 10.1038/s41598-025-88316-6

**Published:** 2025-02-12

**Authors:** Lioua Kolsi, Iman Behroyan, Moustafa S. Darweesh, Badr M. Alshammari, T. Armaghani, Meisam Babanezhad

**Affiliations:** 1 Department of Mechanical Engineering, College of Engineering, University of Ha’il, Ha’il City, 81451 Saudi Arabia; 2 Faculty of Mechanical and Energy Engineering, Shahid Beheshti University, Tehran, Iran; 3 Civil Engineering Department, College of Engineering, Northern Border University, P.O. Box 1321, Arar, Saudi Arabia; 4 Department of Electrical Engineering, College of Engineering, University of Ha’il, Ha’il City, 81451 Saudi Arabia; 5 Department of Engineering, West Tehran Branch, Islamic Azad University, Tehran, Iran; 6 Department of Mechanical Engineering, National University of Skills (NUS), Tehran, Iran

**Keywords:** Artificial intelligence, ANFIS, CFD, Bubble column reactor, Chemical engineering, Mechanical engineering

## Abstract

Air as an inert gas is usually applied for homogenization and mixing liquids. In the current research, we study a 3-D bubble column reactor (BCR) filled with water by using an Artificial intelligence algorithm (AI) and CFD. We used one of the adaptive networks and fuzzy inference systems (ANFIS) to study fluid flow and see its effect on the accuracy of the AI. Therefore, the Gaussian membership function was used to have a prediction in the 3-D BCR. Also, the grid partition system was used to cluster the data. The number of membership functions increases in the training process of the AI system, from 2 to 5. The influence of input numbers on AI data prediction is analyzed. The four inputs in the training process included air velocity and pressure, as well as the x-direction and z-direction. Finally, air vorticity was considered as the output parameter of the study in the predictions. Correlations were developed to predict the air vorticity in each node using x and z direction, air velocity, and pressure. The results showed the AI accuracy increased by the rise of membership and input numbers. The AI intelligence level was found by five memberships and four inputs. The AI and CFD were in suitable agreement (regression number around 1). The developed correlations could simplify the calculation of air vorticity instead of using the complicated and time-consuming CFD simulation. As far as the authors know, there are no studies that have developed correlations to find the air vorticity in bubble column reactors.

**Nomenclature**.

**Roman symbols**.

$$\:{\text{M}}_{\text{I}}$$ Interfacial force,$$\:\left(\text{N}\right)$$

$\:{\text{M}}_{\text{T}\text{D}}$ Turbulent dispersion force,$\:\left(\text{N}\right)$

$$\:{\text{M}}_{\text{D}}$$ Drag force,$$\:\left(\text{N}\right)$$ 

$$g$$ Gravitational acceleration,$$(\text{m}/\text{s}2\:)$$ 

$k$ Turbulent kinetic energy,$({\text{m}}^{2}/{\text{s}}^{2})$ 

$$p$$ Static pressure,$$({\text{N}}/{\text{m}}^{2})$$ 

$\:{\text{u}}_{k}$ bubble or liquid velocity,$(\text{m}/\text{s})$

Greek Symbols.

$\:{\in\:}_{k}$ bubble or liquid volume fraction

$\:\epsilon\:$ Dissipation rate of turbulence kinetic energy,$\:({\text{m}}^{2}/{\text{s}}^{3})$

Subscripts.

$\:G$ gas/bubble phase

$$\:L$$ Liquid phase

## Introduction

Feeding air in a cylindrical water reservoir is applied as a tool for mixing and homogenizing hydrodynamics parameters. A two-phase bubble column is a multiphase reactor with a gas phase spread in a liquid phase as the “coalescence-induced” or dispersed bubbles. An interface separates the two phases in which the interfacial transport phenomena might happen. A vertical cylinder is included in the simplest bubble column configuration where the gas is inserted at the bottom via a gas sparger while supplying the liquid phase in a batch manner. However, it might result in counter-currently or co-currently toward the upward gas stream. In spite of the simple bubble column plan, coupling, and multifaceted fluid dynamics contacts exist within the phases, revealing in the dominant flow trends^1^.

A bubble column has many benefits, including decent mass and heat transfer, ease of operation, no moving parts, and low maintenance and operating expenses. Back mixing is the main shortcoming of bubble column reactors, adversely affecting product conversion. Momentum is conveyed in these reactors from the faster gas phase moving upward to the slower liquid or slurry phase. The acting liquid superficial velocity (within 0–2 cm/s) is slower compared to the superficial gas velocity (1–50 cm/ s). Therefore, the gas flow mainly controls such as reactors’ hydrodynamics. Mostly, two flow regimes exist in the bubble columns with relatively big diameters, i.e., heterogeneous and homogeneous^2^.

The numerical methods have been utilized so far in numerous investigations for predicting the bubble column reactors as a result of some problems still existing in scaling up and designing the bubble columns^3–5^. Through computational fluid dynamics (CFD), our partial information regarding the complex hydrodynamic procedures happening in the bubble column is enhanced^6^. There are several models for interphase turbulence and forces in the literature^7–10^. For example, Gupta et al. studied the lift force, turbulent dispersion drag force and added mass model^3,11,12^. Moreover, various turbulence models such as the standard k–ɛ model, Large Eddy Simulation (LES), and Reynolds Stress Model were utilized for forecasting flow patterns into the bubble column^13–16^. RSM and LES models are favored among existing turbulence models as a result of a more precise prediction of the gas hold-up, swirling flow, and velocity into the bubble column. However, using the LES model can be very time-consuming and expensive, and we can not use this model during optimization or scale-up of the bubble column reactor. Recently many studies^17,18^ have been developed for prediction of bubble column reactors with a combination of CFD and AI with significantly less amount of computational time. This tool is a promising framework for scale-up and optimization of the bubble column reactor.

Currently, adaptive neural network-based fuzzy inference system (ANFIS) was run to simulate the flow pattern within bubble column reactors. Pourtousi et al.^19^utilized the information regarding the hydrodynamics of the multiphase reactor for the training stage. They indicated that the CFD combined with ANFIS is an excellent instrument for estimating BCR behavior. It was found that the ANFIS algorithm is a proper technique instead of CFD approaches for simulating bubble flow into the BCR for the homogeneous flow regime, indeed with almost equal bubbles with velocity and spherical shape. They specifically model the reactor with different sparger specifications, such as sparger diameter, and they suggested a new framework to optimize the process without participating in the CFD method during optimization. This algorithm has been extended by different researchers, and they optimize the AI by tuning AI parameters^20,21^. For example, Babanezhad et al.^22^ found that the number of input parameters can significantly change the accuracy of the AI method and time of training of the CFD data set. However, there is still a question about the impact of functions on the accuracy of the method when the number of input and membership functions is changing.

There is no study to predict the air vorticity in bubble column reactors using CFD and AI learning. The value of air vorticity could help engineers and designers for finding the mixing and disturbance in bubble reactors. In this study, the Difference between two sigmoidal membership functions (dsigmf) was used in the training stage, and four inputs, including x-direction, z-direction, air velocity, and pressure, and one output (air vorticity), were used during training mode. Five membership functions were used to study the system’s accuracy with different patterns of inputs. Data clustering in the system was a grit partition that was used together with the membership function. We also used local node comparison for the data set to compare the AI domain with the CFD domain as a pattern recognition method. After finding the intelligence level of the AI algorithm, correlations are developed for the first time to find the air vorticity as a function of air and pressure for each node.

## Method

### CFD method

The two-phase model was designed based on the Eulerian-Eulerian approach and was utilized to simulate the gas-liquid interaction using ANSYS CFX^23^. This approach considers each phase as a continuum in the domain. Ensemble-averaged mass and momentum transport equations for each phase are the bases of the Eulerian modelling framework functions.

Continuity equation:

1 $$\:\frac{\partial\:}{\partial\:\text{t}}\left({{\uprho\:}}_{\text{k}}{\epsilon\text{}}_{\text{k}}\right)+\nabla\:\left({{\uprho\:}}_{\text{k}}{\epsilon\text{}}_{\text{k}}{\text{u}}_{\text{k}}\right)=\:0$$

Momentum transfer equation:

2 $$\:\frac{\partial\:}{\partial\:\text{t}}\left({{\uprho\:}}_{\text{k}}{\epsilon\text{}}_{\text{k}}{\text{u}}_{\text{k}}\right)+\nabla\:\left({{\uprho\:}}_{\text{k}}{\epsilon\text{}}_{\text{k}}{\text{u}}_{\text{k}}{\text{u}}_{\text{k}}\right)=-\nabla\:\left({\epsilon\text{}}_{\text{k}}{{\uptau\:}}_{\text{k}}\right)-{\epsilon\text{}}_{\text{k}}\nabla\:\text{p}+{\epsilon\text{}}_{\text{k}}{{\uprho\:}}_{\text{k}}\text{g}+{\text{M}}_{\text{I},\text{k}}\:\:\:\:$$

The interphase drag force and turbulent dispersion force rule the total interfacial force between the two phases, which is:

3 $$\:{\text{M}}_{\text{I},\text{L}}=-{\text{M}}_{\text{I},\text{G}}={\text{M}}_{\text{D},\text{L}}+{\text{M}}_{\text{T}\text{D},\text{L}}\:\:\:\:\:\:\:$$

Tabib et al. gave a detailed description of interfacial force models used in the present study^11^. In addition to the interfacial forces, the turbulence model is among the key factors that represent the hydrodynamic properties of the bubble column. The K–ε model has been widely used in the past two decades to explain the flow pattern in the bubble columns. The model is economical and reliable enough, given its smile design and low computational needs. Here, the turbulence model was used for the whole simulation. The parameters of the model were the same as those of Pourtousi et al.^24^.

#### Geometrical structure

**Fig. 1:**
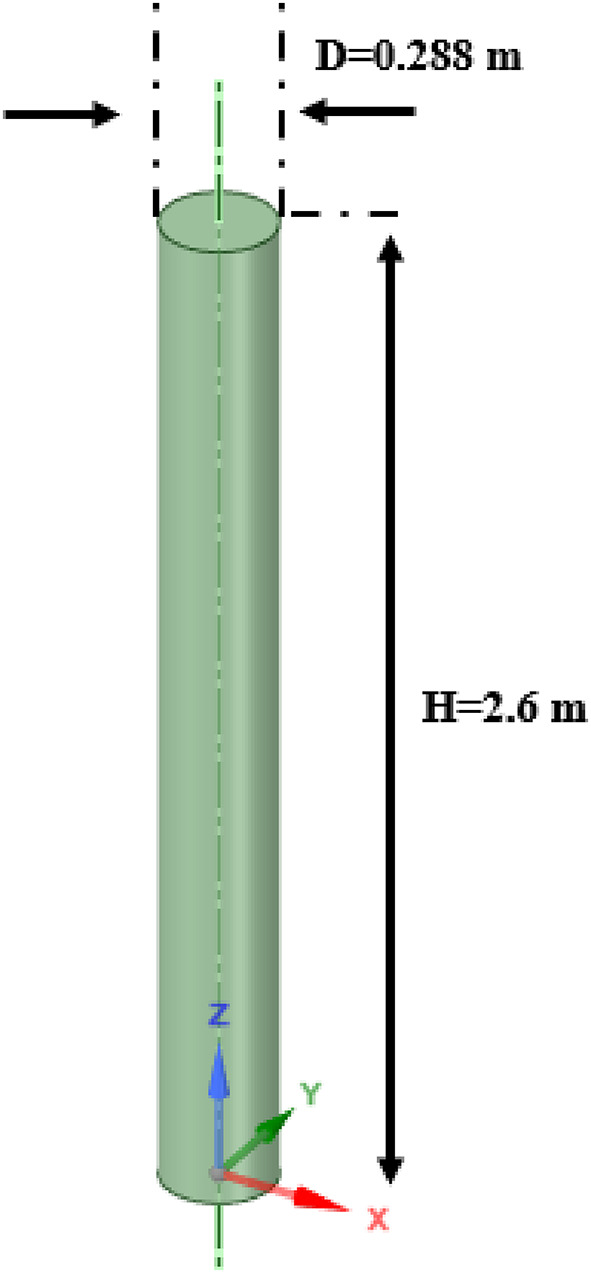
Geometry of case study.

Following Pfleger and Becker^13^, a cylindrical bubble column reaction, 2.6 m in height and 0.288 m in diameter was used (see Fig. 1). The superficial velocity of the gas was 0.008 m/s at room conditions. Further information about boundary conditions (e.g., walls and outlet pressure) is available in Pfleger and Becker^13^. Moreover, the boundary condition of the inlet is the same as that of Tabib et al.^11^.

Figure 1.

#### Grid

**Fig. 2:**
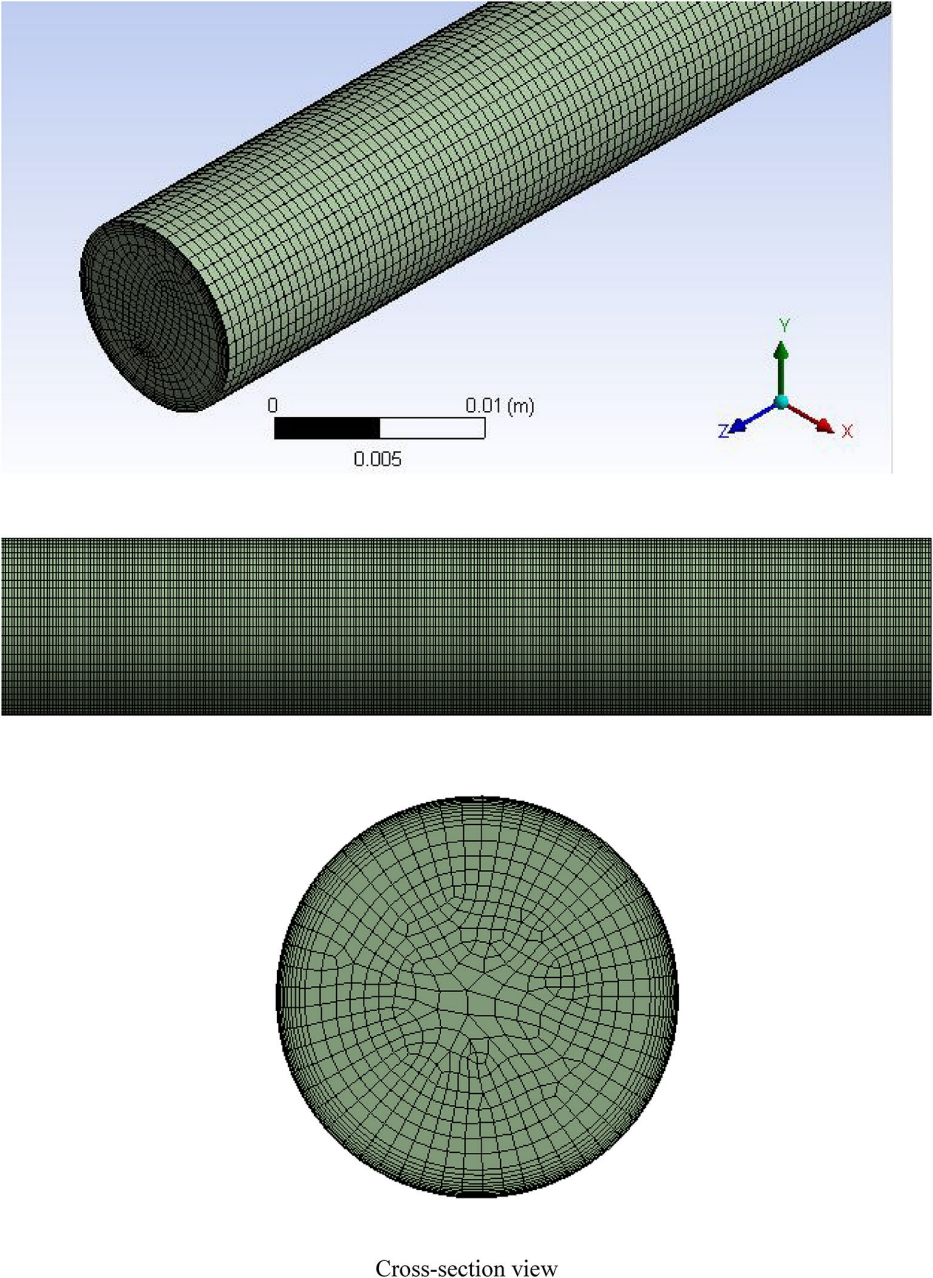
Grid structure of case study.

**Fig. 3:**
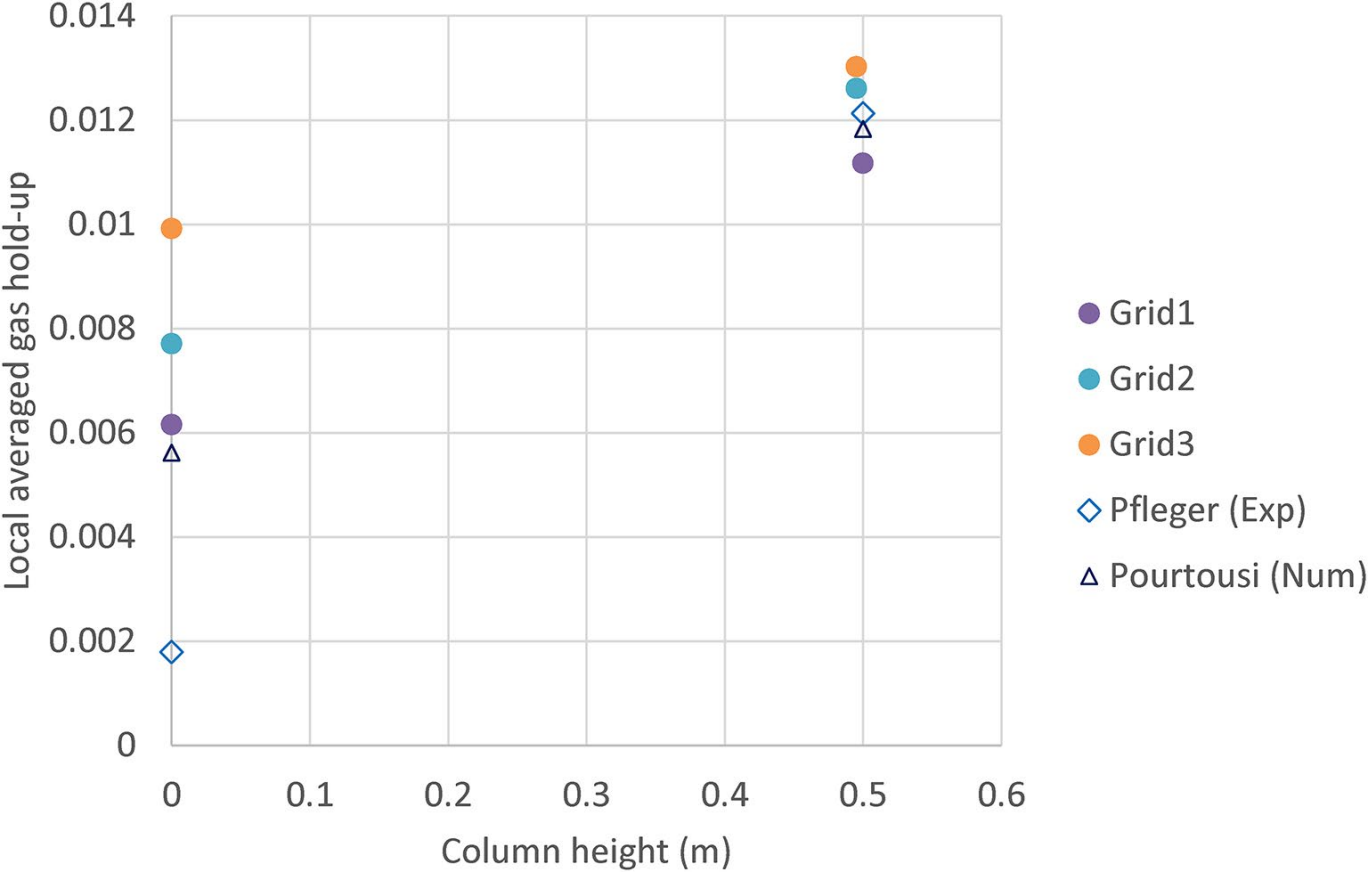
CFD grid independency test for three grid sizes; Comparison of local gas hold-up from CFD results (Grids 1, 2, and 3), experimental data from Pfleger and Becker^13^, and data from Pourtousi et al.^25^, measured at heights 0 to 0.5 m with a bubble diameter of 4 mm.

Throughout the domain, as shown in Fig. 2, a structured grid designed based on a hexahedral grid was utilized. Comparison of local gas hold-up from CFD results (Grids 1, 2, and 3), numerical data from Pfleger and Becker^13^, and data from Pourtousi et al.^25^, measured at heights 0 to 0.5 m with a bubble diameter of 4 mm has been done (see Fig. 3). Among several grid sizes, the results show that the number of 1,250,000 control volumes (grid 1) is needed for mesh-independent results.

presents a comprehensive validation study comparing the relationship between gas hold-up and superficial gas velocity across multiple research works. The mathematical correlation developed by Joshi & Sharma^26^is represented by a solid blue line, showing a nearly linear increase in gas hold-up with superficial gas velocity. This trend is corroborated by both experimental (triangular markers) and numerical (square markers) data from Pfleger & Becker^13^, which demonstrate good agreement with the theoretical prediction, particularly at lower velocities (0–0.01 m/s), with slight experimental deviations at higher velocities (0.015–0.02 m/s). The more recent numerical simulation by Pourtousi et al.^27^, depicted by a red line with circular markers, aligns remarkably well with both the historical correlation and Pfleger & Becker’s data, validating the consistency of computational approaches over time. The current study’s results, represented by multiple numerical simulation points, fall precisely along these established trends, particularly matching the numerical predictions of previous studies, thereby demonstrating the reliability and accuracy of the present computational approach in predicting gas hold-up behavior in bubble column systems. Additionally, the current study shows the linear relationship between flow rate and gas bubble concentration in bubble columns, which is in strong agreement with previous experimental, mathematical, and numerical studies.

According to Table 1, the MAE error approaches zero in comparison with Pourtousi et al.‘s^27^study, while showing an approximate value of 0.003 when compared to the mathematical correlations of Pfleger and Becker^13^and Joshi and Sharma^26^.

**Table 1:** MAE error for validation test.

Pourtousi et. Al[27]	1E-06
Joshi & Sharma[26] Pfleger & Becker[13]	0.0028321

Figure 4.

### ANFIS (Adaptive network fuzzy inference system)

The network structure of ANFIS includes two sectors known as the consequence and premise parts. Training ANFIS indicates determining the parameters related to these parts using an optimization algorithm. Through ANFIS, the present input-output data pairs are used overtraining, IF-THEN fuzzy rules are then attained to connect these parts^28^.

The schematic of ANFIS’s structure includes five layers, which are provided in Fig. 5. The ANFIS structure involves two inputs and one output, as well as four rules and four membership functions. Based on the ANFIS structure in the figure, the layer structure of ANFIS is clarified in the following.

Figure 5.

**First Layer**.

This section is known as the fuzzification layer, which utilizes membership functions to attain fuzzy clusters from input values. The parameters, such as the type of membership function along with these parameters, are known as the premise parameter^29^. To calculate each membership function’s membership levels, these parameters are used, as provided in (4) and (5). µ_ij_ represents the membership levels attained from this layer and a_ij1,_ c_ij1,_ a_ij2,_ and c_ij2_ are dsigmf constant parameters where X_i_ indicates inputs data (X_1_ = x-direction, X_2_ = C-direction, X_3_ = V_g_, X_4_ = P), *i* is an indicator of inputs (1 to 4) and *j* is the number of input MFs (1 to 5).

4 $$\:{\mu\:}_{ij}= dsigmf(x_i; a_ij1, c_ij1, a_ij2, c_ij2)=\:\:\frac{1}{1+{e}^{-{a}_{ij1}\left({X}_{i}-{c}_{ij1}\right)}}-\frac{1}{1+{e}^{-{a}_{ij2}\left({X}_{i}-{c}_{ij2}\right)}}$$

5 $$\:{O}_{ij}^{1}=\:{\mu\:}_{ij}(x_i)$$

**Second Layer**.

This layer is known as the rule layer. Firing strengths (w_k_) for the rules are created utilizing membership values calculated in the fuzzification layer. Multiplication of the membership values results in w_k_ values as follows.

6 $$\:{O}_{k}^{2}={\omega\:}_{k}=\:{\mu\:}_{1j}(x)\:\:{\mu\:}_{1j}(z)\:\:{\mu\:}_{3j}(v_g)\:\:{\mu\:}_{4j}(p)k=1\,\, to\,\,625$$

**Third Layer**.

This layer is termed the normalization layer, which determines normalized firing strengths related to each rule. The normalized value is the ratio of the firing strength of the *k*^th^ rule to the overall firing strengths, as provided in (7).

7 $$\:{O}_{k}^{3}=\stackrel{-}{{\omega\:}_{k}}=\:\frac{{\omega\:}_{k}}{\sum\:{\omega\:}_{k}}\:\:\:\:\:\:k=1\:to\:625\:\:\:\:\:\:\:\:$$

**Fourth Layer**.

This layer is known as the defuzzification layer, where weighted values of rules are determined in each node of this layer as in (8). The first-order polynomial is used to calculate this value.

8 $$\:{O}_{k}^{4}=\stackrel{-}{{\omega\:}_{k}}\:{f}_{k}\:k=1\:to\:625\:\:\:\:\:\:\:\:$$

$\:\stackrel{-}{{\omega\:}_{k}}$represents the normalization layer output^30^.

**Fifth Layer**.

It is termed the summation layer, where the real output of ANFIS is attained by summation of the outputs attained for each rule within the defuzzification layer.

9 $$\:{O}_{k}^{5}=\:\sum\:_{k}\stackrel{-}{{\omega\:}_{k}}\:{f}_{k}=\:\frac{{\sum\:}_{k}{\omega\:}_{k}\:{f}_{k}}{{\sum\:}_{k}{\omega\:}_{k}}\:\:\:\:\:\:$$

## Results

For the prediction of the fluid in the BCR, we used the dsigmf membership function for the training process. This membership function was studied with various inputs and membership functions to see how the system sends its intelligent signals.

The flowchart in Fig. 6 delineates a methodical approach to utilizing ANFIS for predicting air vorticity surfaces from CFD data, encompassing the selection of critical fluid characteristics (x and z direction, air velocity, and pressure) as ANFIS inputs, the implementation of grid partition clustering for generating the primary Fuzzy Inference System (FIS) and defining input membership functions, the meticulous setting of FIS and clustering parameters (including data allocation for training, iteration counts, and membership function specifications), followed by an iterative process of ANFIS training, error recording, and strategic adjustments to input variables and membership functions, culminating in the selection of the most intelligent model based on error minimization and determination coefficient maximization, which is then validated and applied to predict air vorticity surfaces across diverse input scenarios.

Figure 6.

The bubbles are created from the sparger orifices by using the air pressure inserted into its sparger and the pressure difference in the BCR. The flow can have different forms, such as homogeneous or heterogeneous. If the flow is in the former form, we can see the bubbles that have similar sizes and insignificant interactions with each other, but the latter flow has significant interaction among bubbles, meaning that the amount of merging and breaking up is high. Due to turbulence flow inside the BCR, the pieces of bubbles can have different sizes.

In the current research paper, we study a homogenous flow; therefore, the complexity of the flow in the study is not high. Moreover, we can see how we can perform an AI system entirely in the BCR. Firstly, the BCR could be studied via CFD methods, and the solving is possible through final volume methods designed in parallel. After solving and post-processing the CFD-created data, we could create data sets that are the input and output matrixes, enabling us to have input and output for the data sets. By having the data sets originate from input and output data, we could create a training domain. In the current research, we used the dsigmf function for the training process by using different inputs and membership functions. The errors that originate from AI and CFD could be compared to see the differences in the fluid flow in the AI and CFD domains. For the training process, we used 70% of the data, and for testing the data, the remaining 30% plus the used 70% in the training process was used for comparison of the data in AI and CFD. For the iterative algorithm, we had about 200 steps, and the AI system reached a kind of convergence, meaning that we did not need training for the system anymore. Furthermore, the dsigmf function was used in training, and for data clustering in the system, grid partition was used together with the membership function. As mentioned earlier, we used up to 5 membership functions for studying the accuracy of the system, and we applied four inputs for the training of the system, which are x-direction, z-direction, air velocity, and pressure in the BCR. Moreover, the only output of the study is the vorticity of air.

As shown in Fig. 7a, after considering 70% of the data for the training process, the data was compared to CFD. The purpose was to see the amount of error between the AI and CFD data by the calculation of the regression number (R). As shown in the figure, the different numbers of membership functions were used, including 2, 3, 4, and 5. Figure 7a was considered with two numbers of inputs. As shown in the figure, when there are two numbers of membership functions, the regression number (R) is around 0.65, meaning that the predicted value deviates from the real one. By increasing the membership functions to 5, the measure of R approaches 0.98. This means that by increasing the number of membership functions, the predicted data are closer to the CFD data with less deviation.

Figure 7b shows the testing of the data. The remaining percent of the data (i.e.,30%) and 70% of the data used in training is considered in the testing by using CFD. As the training process, the evaluation is done for different membership functions and two inputs. Similar to the training process, the amount of R increases from 0.68 to 0.98.

Figure 7a and b.

As shown in Fig. 8a, the inputs increased to see the changes in the accuracy of the system. In the training process, the inputs increased to 3, and similar to the previous results, the membership functions were considered from 2 to 5. As shown in the figure, when the number of inputs increased to 3, the value’s R increased slowly from 0.95 to 0.998. The same procedure was done for the testing as well, and as seen in Fig. 8b, the AI system overlapped the training results (regression increases from 0.95 to 0.997).

Figure 8a and b.

In Fig. 9a and b, the number of inputs increased to 4, and as said earlier, input 4 was air velocity, which was also added to the system. When one input was added to the system in the training process, the system suddenly showed intelligence and sent its intelligent signals.

Figure 9a and b.

As shown in the Figure, at the beginning of the process, the regression is very high compared to previous conditions (0.96). Nevertheless, we can still train the system better, and therefore, the system can have better accuracy. By increasing the number of membership functions up to 5, the system’s amount of regression increases significantly (roughly 1). Furthermore, the number of elements whose error was low increased, and therefore, the system became very intelligent. In the testing domain, the same procedure was used, and the same results were achieved in both testing and training processes (the regression is around 0.996). Generally, when the testing and training processes overlapped, we could make sure that the training system was suitable because two different procedures were evaluated and compared, so we could ensure that the system had a suitable capacity.

In Fig. 10, a 3-D plot was used to answer some questions. First, how do CFD and AI data overlap each other? Second, do they completely overlap each other? And finally, do they have some differences? The purpose behind them was to find out how the local data matches with its target value and what the differences are. This condition was considered the best intelligence of the system when the number of inputs was four and the membership function was five. Figure 10 shows that after the training and testing processes, the data was used in the prediction process; consequently, some of the points were not predicted accurately compared to the target point however, these single points related to the boundary condition. The same procedure was done for a variety of inputs. It is worth mentioning that this condition referring to the distance of the local points is closely related to the boundary condition of the system, and we need to introduce and change it in CFD and AI system; therefore, we can consider the error of the system. It is also worth mentioning that in AI, there is no description or boundary condition because AI does not have any understanding of our data set. We need to describe and consider those single local points in AI, which can be done by filtering or boundary condition descriptions in the AI domain.

Figure 10.

Finally, Fig. 11 shows the general prediction of the system to see the differences in internal patterns in the system in CFD and AI domains. As shown in the Figure, the complex data set process in CFD can be predicted by ANFIS, and all the complexity and the relationship among the points could be found by AI when the number of input parameters was four and the number of membership functions was five. This point was when the AI sent its intelligent signals, and the system could be used in different boundary conditions.

**Figure 11**.

Equation ʄ 10 states that neither the dsigmf parameters nor the resulting parameters are known for each cluster. The number of parameters could be found at the highest level of ANFIS intelligence.

The dsigmf function and its parameters are displayed in Table 2. Table 3 lists the dsigmf parameters at the highest level of ANFIS intelligence. Additionally, Table 4 provides the constant parameters for each of the output’s 625 membership functions, which are constant membership function types. Consequently, all of the parameters required for Eq. ʄ 10 have been acquired. The Air Vorticity as a function of x, z, Vg, and P is provided by the developed equation.

**Table 2:** Mathematical formula of difference between two sigmoidal membership functions used in this work.

Membership Function	Formula
Dsigmf	$\:{\mu\:}_{i}=\frac{1}{1+{e}^{-{a}_{1}\left(x-{c}_{1}\right)}}-\frac{1}{1+{e}^{-{a}_{2}\left(x-{c}_{2}\right)}}$

**Table 3:** Inputs membership functions (dsigmf) parameters in ANFIS intelligence learning process.

i (input)	j (MFs)	aij1	cij1	aij2	cij2
1	1	174.216	−0.180	174.216	−0.070
1	2	174.216	−0.096	174.216	−0.007
1	3	174.216	−0.027	174.216	0.053
1	4	174.216	0.031	174.216	0.106
1	5	174.216	0.088	174.216	0.179
2	1	173.611	−0.180	173.611	−0.095
2	2	173.611	−0.118	173.611	−0.010
2	3	173.611	−0.047	173.611	0.053
2	4	173.611	0.029	173.611	0.102
2	5	173.611	0.077	173.611	0.180
3	1	59.088	−0.086	59.088	0.166
3	2	59.088	0.148	59.088	0.381
3	3	59.088	0.390	59.088	0.597
3	4	59.088	0.519	59.088	0.813
3	5	59.087	0.706	59.088	0.972
4	1	2.000	−619.125	1.996	−612.871
4	2	2.000	−612.875	1.998	−606.621
4	3	1.995	−606.627	2.000	−600.375
4	4	2.000	−600.375	2.000	−594.126
4	5	1.998	−594.128	2.000	−587.875

**Table 4:** ANFIS method constant parameters while output membership functions are constant.

k	F_k_		k		F_k_		k		F_k_		k		F_k_		k		F_k_		k		F_k_		k		F_k_		
1	−9.9E-04		46		−4.9E-11		91		3.6E-02		136		1.0E-05		181		5.8E + 00		226		2.3E + 01		271		−9.9E-10		
2	−1.2E + 02		47		−1.4E-07		92		−1.5E-02		137		−1.7E-03		182		5.8E + 00		227		4.2E + 00		272		−3.7E-05		
3	1.6E + 01		48		−4.3E-07		93		1.2E-03		138		−5.2E-02		183		1.9E + 01		228		−6.5E-01		273		8.0E-05		
4	4.3E-02		49		−7.9E-09		94		6.4E-07		139		−6.0E-06		184		−1.9E-02		229		−2.4E-06		274		−1.8E-05		
5	1.6E-07		50		−1.5E-14		95		−3.8E-11		140		−2.3E-11		185		−7.4E-08		230		−9.2E-12		275		−4.5E-10		
6	−2.2E-04		51		4.3E + 00		96		3.2E-05		141		1.8E-08		186		1.1E + 01		231		5.4E + 00		276		6.7E + 00		
7	−3.1E + 01		52		2.9E + 00		97		1.5E-04		142		−8.0E-06		187		4.0E + 00		232		5.2E + 00		277		5.3E + 00		
8	−1.4E + 00		53		4.6E + 00		98		1.7E-05		143		−2.4E-05		188		−1.7E + 01		233		1.1E + 01		278		6.2E + 00		
9	8.1E-03		54		1.7E-01		99		4.5E-07		144		−5.8E-08		189		2.5E-01		234		7.0E-05		279		7.0E + 00		
10	3.1E-08		55		6.6E-07		100		−1.3E-10		145		−2.0E-13		190		4.5E-06		235		2.7E-10		280		2.7E-05		
11	2.8E-09		56		−7.5E + 00		101		−1.3E + 01		146		−1.3E-12		191		−5.9E + 01		236		−8.4E + 00		281		1.4E + 01		
12	5.9E-04		57		−7.5E-01		102		5.2E + 00		147		−4.3E-09		192		4.5E + 00		237		5.4E + 00		282		6.3E + 00		
13	1.4E-02		58		−7.2E-01		103		−4.3E + 00		148		−1.1E-08		193		3.2E + 01		238		4.7E + 00		283		7.3E + 00		
14	1.4E-06		59		4.1E-01		104		−1.6E-05		149		−4.5E-10		194		1.2E + 00		239		3.8E-01		284		−9.6E + 00		
15	5.2E-12		60		1.6E-06		105		−6.1E-11		150		−1.2E-14		195		7.0E-06		240		8.6E-07		285		−3.7E-05		
16	1.6E-12		61		−1.7E-01		106		1.3E + 00		151		9.6E + 00		196		−1.2E-02		241		−7.5E + 01		286		−1.8E + 01		
17	3.0E-07		62		−1.1E + 00		107		4.9E + 00		152		4.1E + 00		197		−4.7E + 00		242		8.5E + 00		287		1.0E + 01		
18	6.9E-06		63		−1.7E-02		108		2.2E + 00		153		3.5E + 00		198		1.9E + 00		243		−1.2E + 01		288		8.0E + 00		
19	6.8E-10		64		5.2E-06		109		8.1E-06		154		7.1E + 00		199		7.6E-03		244		3.3E-01		289		−9.9E + 00		
20	2.6E-15		65		8.6E-11		110		3.1E-11		155		2.7E-05		200		−1.3E-07		245		−8.4E-05		290		−1.7E-05		
21	−3.3E-18		66		−1.7E-03		111		3.0E + 00		156		1.0E + 01		201		6.4E + 00		246		1.2E + 00		291		−9.6E-01		
22	4.7E-12		67		4.4E-03		112		3.0E-01		157		2.5E + 00		202		6.1E + 00		247		−2.6E + 00		292		8.2E + 00		
23	1.1E-10		68		1.9E-03		113		−1.0E-02		158		2.2E + 00		203		3.1E + 01		248		2.8E + 00		293		7.6E + 00		
24	2.6E-15		69		2.1E-05		114		7.0E-06		159		2.0E + 00		204		1.2E-04		249		6.8E-04		294		−2.6E + 01		
25	−1.7E-19		70		1.3E-10		115		1.6E-11		160		7.7E-06		205		4.5E-10		250		−6.6E-06		295		−6.0E-04		
26	1.2E + 02		71		−2.3E-07		116		4.0E-03		161		3.3E + 01		206		6.2E + 00		251		9.3E-02		296		−2.1E-03		
27	2.0E + 00		72		−8.8E-05		117		1.5E-02		162		9.6E + 00		207		6.5E + 00		252		4.1E + 00		297		−6.6E + 01		
28	4.4E + 00		73		3.5E-05		118		−1.0E-03		163		2.6E + 00		208		1.5E + 02		253		2.1E + 00		298		2.6E + 00		
29	1.5E + 00		74		1.4E-07		119		6.1E-06		164		3.7E-03		209		−1.1E-03		254		−5.1E + 00		299		−1.7E + 01		
30	5.6E-06		75		−1.2E-12		120		−1.6E-09		165		2.3E-08		210		−3.9E-09		255		−1.9E-05		300		−3.6E-05		
31	−4.0E + 00		76		3.3E + 00		121		2.2E-05		166		4.7E-02		211		8.0E + 00		256		−6.7E-02		301		9.5E + 00		
32	3.1E + 00		77		3.0E + 00		122		−3.8E-05		167		−7.5E + 00		212		8.0E + 00		257		−1.4E + 02		302		1.1E + 01		
33	2.3E-01		78		−4.1E + 01		123		5.2E-05		168		5.9E-01		213		4.4E + 01		258		−4.1E + 00		303		6.8E + 01		
34	−1.6E + 00		79		−3.6E-04		124		1.3E-08		169		3.7E-03		214		−3.7E-01		259		5.6E + 00		304		6.2E-04		
35	−6.1E-06		80		−1.4E-09		125		−1.2E-10		170		6.6E-09		215		−5.1E-07		260		2.1E-05		305		2.4E-09		
36	1.1E-03		81		−5.0E + 00		126		−2.1E-01		171		−2.9E-06		216		3.7E + 01		261		−2.4E-05		306		7.6E + 00		
37	−1.7E-02		82		3.0E + 00		127		−3.4E-02		172		−7.5E-03		217		1.2E + 01		262		1.7E-02		307		9.7E + 00		
38	−2.9E-01		83		8.9E + 01		128		2.8E + 00		173		−2.3E-02		218		−4.8E + 00		263		−3.0E + 00		308		−2.3E + 00		
39	−3.8E-05		84		2.9E-04		129		6.3E-02		174		−4.3E-04		219		4.2E-02		264		−2.7E-02		309		−4.1E-01		
40	−1.5E-10		85		1.1E-09		130		2.4E-07		175		−8.5E-10		220		−2.1E-06		265		−1.0E-07		310		−1.6E-06		
41	1.1E-06		86		−1.2E + 00		131		−2.1E-02		176		4.3E + 00		221		1.6E + 00		266		−1.2E-06		311		8.3E + 00		
42	−1.5E-04		87		−2.3E-02		132		4.9E + 01		177		4.9E + 00		222		6.7E + 00		267		6.0E-04		312		7.2E + 00		
43	−1.4E-04		88		−1.5E-02		133		−2.9E + 00		178		−1.0E + 01		223		9.5E-01		268		−5.2E-02		313		8.0E + 00		
44	5.1E-08		89		−7.2E-06		134		2.4E-02		179		−4.1E-02		224		2.5E-02		269		−2.1E-03		314		1.1E + 02		
45	3.7E-14		90		−1.1E-11		135		9.2E-08		180		−1.6E-07		225		−7.2E-06		270		−7.3E-09		315		5.9E-04		
***k***		**F**_**k**_		***k***		**F**_**k**_		***k***		**F**_**k**_		***k***		**F**_**k**_		***k***		**F**_**k**_		***k***		**F**_**k**_		***k***		**F**_**k**_	
316		3.4E + 01		361		2.7E + 01		406		−7.3E + 00		451		−1.9E-03		496		2.4E-01		541		−9.3E-05		586		−8.4E-04	
317		7.6E + 00		362		5.1E + 00		407		1.3E + 01		452		−4.7E-03		497		−1.1E + 01		542		−7.9E-01		587		−2.1E-03	
318		5.9E + 00		363		5.6E + 00		408		7.6E + 00		453		2.3E-06		498		4.6E + 00		543		−1.0E + 01		588		8.5E-03	
319		−1.9E + 02		364		1.2E + 00		409		−3.4E + 01		454		3.0E-09		499		8.2E + 00		544		−5.4E + 00		589		6.3E-01	
320		2.1E-02		365		−2.1E-02		410		−1.3E-04		455		−5.4E-12		500		−5.3E + 00		545		−1.5E + 01		590		−2.0E + 00	
321		7.7E-01		366		9.9E + 00		411		−9.7E-01		456		−2.0E-01		501		6.7E-05		546		−3.5E-07		591		3.4E-04	
322		9.5E + 00		367		5.1E + 00		412		7.4E + 00		457		−3.0E-01		502		1.5E + 01		547		−6.2E-02		592		−5.9E-01	
323		7.8E + 00		368		5.2E + 00		413		7.4E + 00		458		−5.4E-01		503		5.1E + 01		548		4.6E + 01		593		−8.0E + 01	
324		1.7E + 02		369		2.3E + 01		414		1.1E + 01		459		−1.2E-04		504		−8.8E-02		549		1.6E + 01		594		−4.3E + 00	
325		−6.4E-03		370		−3.4E + 00		415		2.1E-01		460		−1.8E-06		505		−3.4E-07		550		−1.2E + 02		595		−6.6E-01	
326		−1.8E + 01		371		1.9E + 01		416		−1.1E + 00		461		−1.5E + 01		506		1.2E-05		551		−2.1E-05		596		6.4E-05	
327		−5.6E + 01		372		7.2E + 00		417		7.7E + 00		462		2.2E + 01		507		2.9E + 00		552		2.6E-03		597		−1.4E + 00	
328		1.5E-02		373		6.0E-01		418		7.8E + 00		463		1.7E + 01		508		8.1E + 00		553		1.8E-03		598		4.4E + 01	
329		−1.6E-08		374		−5.8E + 01		419		1.6E + 01		464		4.8E + 00		509		1.7E-01		554		7.8E-06		599		7.7E + 00	
330		−7.3E-14		375		−2.6E-01		420		−5.3E + 00		465		−6.0E-01		510		4.9E-07		555		−3.1E-11		600		7.6E + 00	
331		1.0E + 01		376		−8.6E-04		421		−1.1E-03		466		1.6E + 01		511		2.1E-06		556		9.3E-04		601		−6.7E-09	
332		8.7E + 00		377		7.4E + 00		422		−7.9E + 01		467		5.1E + 00		512		5.4E-01		557		3.9E-02		602		−5.9E-10	
333		4.7E + 00		378		3.4E + 00		423		6.4E + 00		468		5.0E + 00		513		7.5E + 00		558		−1.8E + 00		603		3.5E-11	
334		−2.4E-02		379		−1.7E + 01		424		−1.0E + 01		469		5.2E + 00		514		−2.5E + 01		559		6.9E-02		604		7.2E-12	
335		−9.8E-08		380		−6.5E-05		425		−4.4E-01		470		3.6E + 00		515		−9.1E-04		560		−2.1E-05		605		−3.8E-11	
336		5.4E + 00		381		−1.3E-04		426		−4.3E-01		471		9.5E-01		516		6.9E-10		561		−1.8E-03		606		−1.3E-05	
337		9.5E + 00		382		2.4E + 00		427		5.1E + 01		472		5.0E + 00		517		1.9E-04		562		6.1E-01		607		−8.8E-06	
338		6.5E + 00		383		2.5E + 00		428		−3.8E-01		473		1.2E + 00		518		−3.6E + 00		563		2.2E + 01		608		1.1E-05	
339		1.8E + 01		384		9.2E + 00		429		2.1E-03		474		−1.0E + 00		519		−6.5E + 00		564		−1.4E + 00		609		2.3E-06	
340		−4.9E-03		385		3.5E-05		430		7.9E-09		475		6.3E-01		520		−3.3E-04		565		1.7E + 01		610		−1.3E-05	
341		5.2E + 00		386		2.6E-06		431		1.9E + 01		476		−1.4E-04		521		4.1E-13		566		−4.6E-03		611		−1.5E-04	
342		8.4E + 00		387		4.3E-01		432		9.3E + 00		477		−1.2E-05		522		1.2E-07		567		−6.9E + 00		612		−2.2E-04	
343		1.2E + 01		388		5.7E + 01		433		2.3E + 01		478		3.5E-07		523		3.1E-03		568		1.2E + 01		613		−2.4E-01	
344		1.0E + 02		389		1.3E + 00		434		−2.8E + 00		479		1.3E-08		524		−3.4E-03		569		1.3E + 01		614		4.1E-01	
345		−1.1E-01		390		1.6E-05		435		−1.0E-05		480		−1.4E-10		525		−1.0E-04		570		1.1E + 01		615		−4.3E + 00	
346		2.5E + 01		391		−7.3E-07		436		−1.7E + 01		481		−2.7E-01		526		−1.5E-05		571		3.4E-05		616		1.3E-03	
347		7.2E + 00		392		4.3E-03		437		8.6E + 00		482		−1.8E-01		527		−4.2E-01		572		4.0E-01		617		−7.6E-02	
348		4.3E + 00		393		−2.8E + 00		438		1.2E + 01		483		1.1E-01		528		−8.9E + 01		573		−2.4E + 01		618		2.1E + 01	
349		1.7E + 00		394		−5.3E-02		439		4.4E + 00		484		4.4E-03		529		−5.3E-02		574		−6.1E + 00		619		1.5E + 01	
350		−2.8E-01		395		1.8E-05		440		2.0E-01		485		−4.9E-05		530		−2.0E-07		575		−2.0E + 00		620		7.2E + 00	
351		−3.2E + 00		396		−5.1E-10		441		−5.1E + 01		486		−3.1E + 00		531		−3.3E-04		576		−9.2E-08		621		1.0E-05	
352		−2.8E-01		397		−3.9E-05		442		6.6E + 00		487		3.2E + 01		532		5.2E + 00		577		−2.3E-07		622		−3.5E-01	
353		5.8E-04		398		2.1E-03		443		6.8E + 00		488		4.6E + 00		533		3.0E + 00		578		3.0E-09		623		6.3E + 01	
354		3.2E-08		399		6.3E-03		444		7.0E + 00		489		−1.7E + 01		534		−1.3E-01		579		1.8E-11		624		9.2E + 00	
355		−3.3E-14		400		−5.7E-06		445		−4.7E + 01		490		−1.7E + 01		535		−8.1E-05		580		−1.8E-11		625		4.5E + 01	
356		1.5E + 01		401		−2.9E-01		446		3.3E-01		491		2.7E + 01		536		−9.8E-05		581		−9.3E-06					
357		5.4E + 00		402		2.4E + 01		447		5.8E-01		492		3.9E + 00		537		1.3E + 00		582		−1.5E-05					
358		8.3E + 00		403		7.6E + 00		448		1.6E + 00		493		5.9E + 00		538		2.1E + 00		583		−2.9E-05					
359		1.0E-02		404		−1.1E + 00		449		1.4E + 00		494		5.9E + 00		539		1.2E + 01		584		1.6E-06					
360		−1.4E-08		405		−4.2E-06		450		1.5E + 02		495		3.2E + 00		540		7.2E + 00		585		−5.9E-06					

10 $$\:Air\:\:Vorticity=\sum\:_{i=1}^{625}\left[\frac{\left({\mu\:}_{1j}\right(X)\times\:{\mu\:}_{2j}(Z)\times\:{\mu\:}_{3j}({V}_{g})\times\:{\mu\:}_{4j}(P\left)\right)}{\sum\:_{m=1}^{625}{\omega\:}_{k}}\:\times\:\:{f}_{k}\right]$$

After developing these equations, CFD modeling is no longer necessary for air vorticity predictions. This approach reduces both time and computational costs, leading to simplified engineering design and development.

## Conclusion

In the current research paper, we studied a 3-D BCR by using CFD calculation. The BCR made a homogenous flow, leading to similar bubbles without any merging (coalescence), break up, and low rate of bubble interactions in the BCR. The AI learning method was used to map CFD generated data set. After mapping the CFD data, relations could be developed between different fluid dynamics parameters. The adaptive networks and Fuzzy functions (ANFIS) were selected for the AI learning. The Gaussian membership function and the grid partition system were used to cluster the data. The sensitive analysis was done by increasing the number of membership functions and inputs. The x-direction, z-direction, air velocity, and pressure are inputs. Air vorticity was considered the output parameter for finding water disturbances. Correlations were developed to predict the air vorticity in each node using x and z direction, air velocity, and pressure.

The results showed that AI accuracy increased with the rise of membership and input numbers. The AI intelligence level was found by five memberships and four inputs. The AI and CFD were in suitable agreement (regression number around 1). The developed correlations could simplify the calculation of air vorticity instead of using the complicated and time-consuming CFD simulation. As far as the authors know, there are no studies to develop correlations to find the air vorticity in bubble column reactors.

### Future study

This work is the study of machine learning applications to predict air vorticity using CFD simulation data. The ANFIS setup could be changed by fluctuating the physical boundary conditions and assumptions. In this study, it was aimed to correlate some parameters like vorticity to other fluid dynamics parameters such as pressure and air velocity. Future investigations are needed to use AI machine learning by considering the other fluid dynamics parameters such as bubble size effect and influence of interfacial forces in a bubble column reactor.

**Fig. 4:**
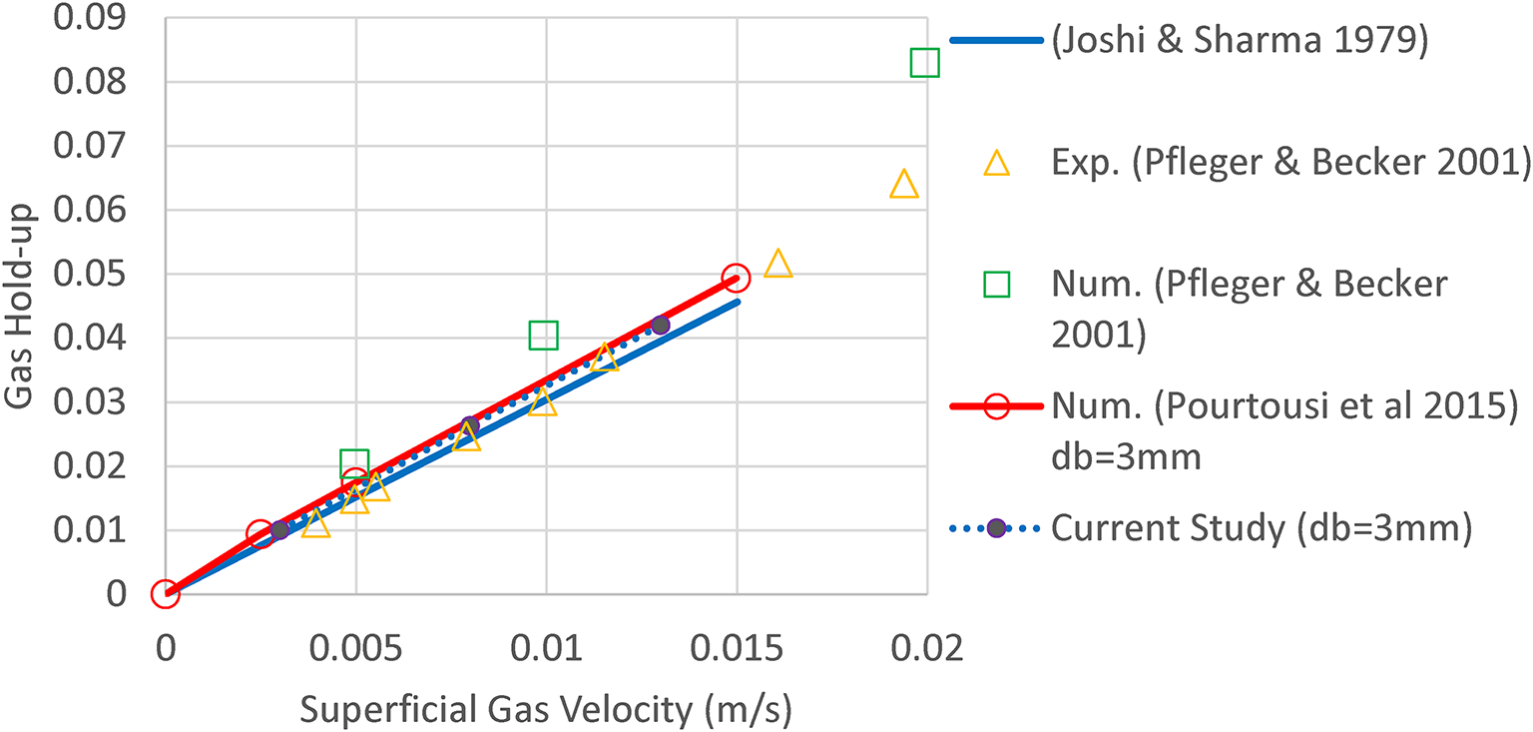
CFD validation; Overall gas hold-up vs. different superficial gas velocity for a bubble diameter of 3 mm, including experimental and numerical data from Pfleger and Becker^13^, the mathematical correlation by Joshi and Sharma^26^, and numerical data from Pourtousi et al.^27^.

**Fig. 5:**
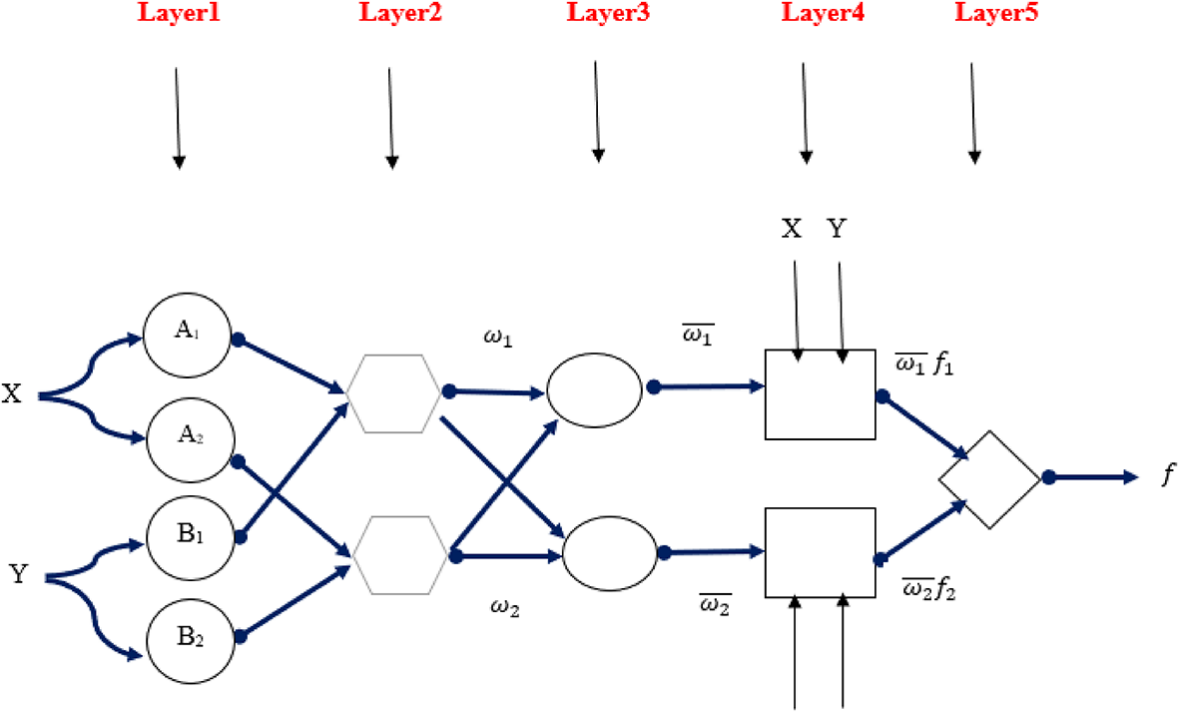
schematic of ANFIS structure for two inputs and two rules.

**Fig. 6:**
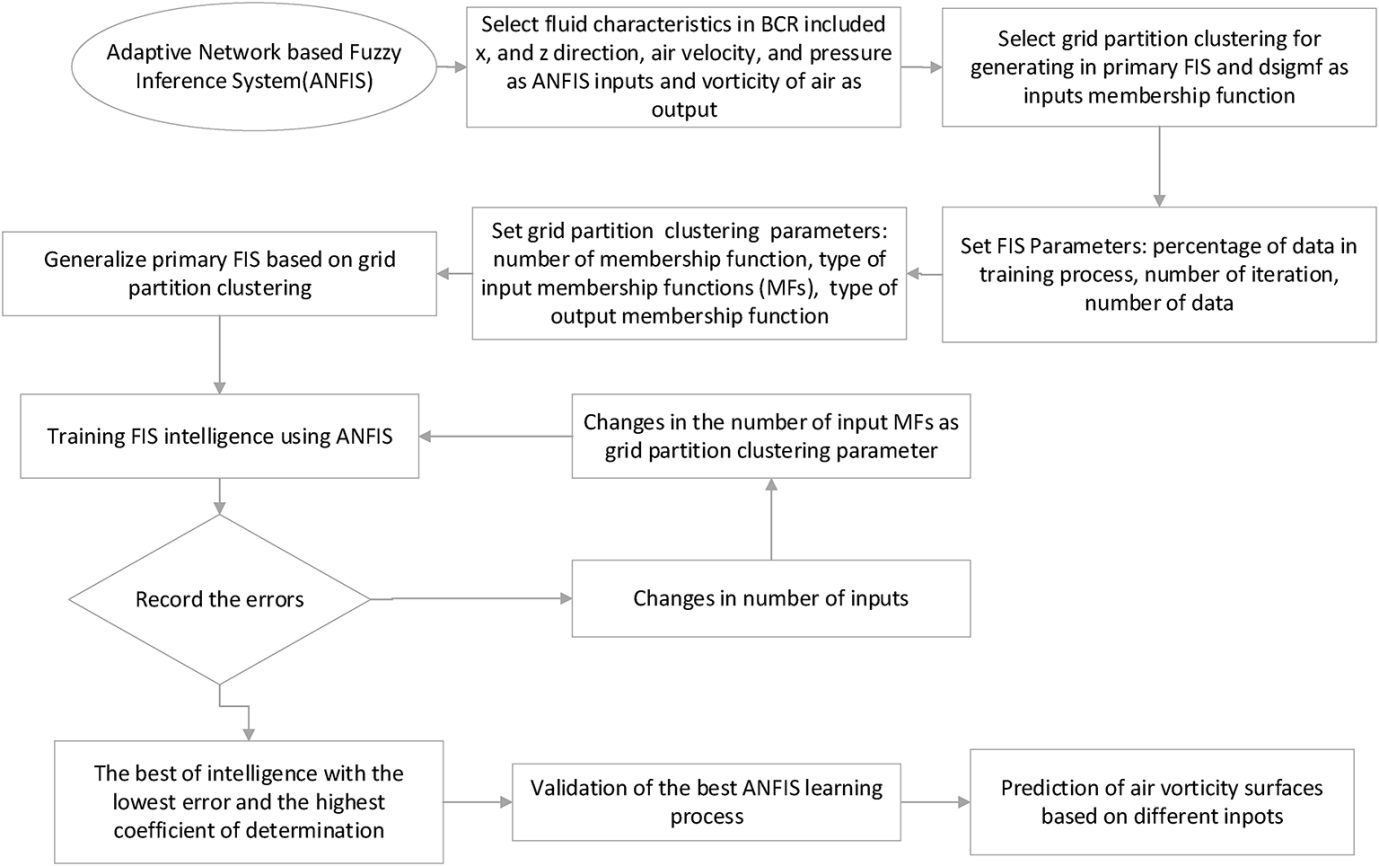
Flowchart of using ANFIS method for predicting CFD result.

**Fig. 7:**
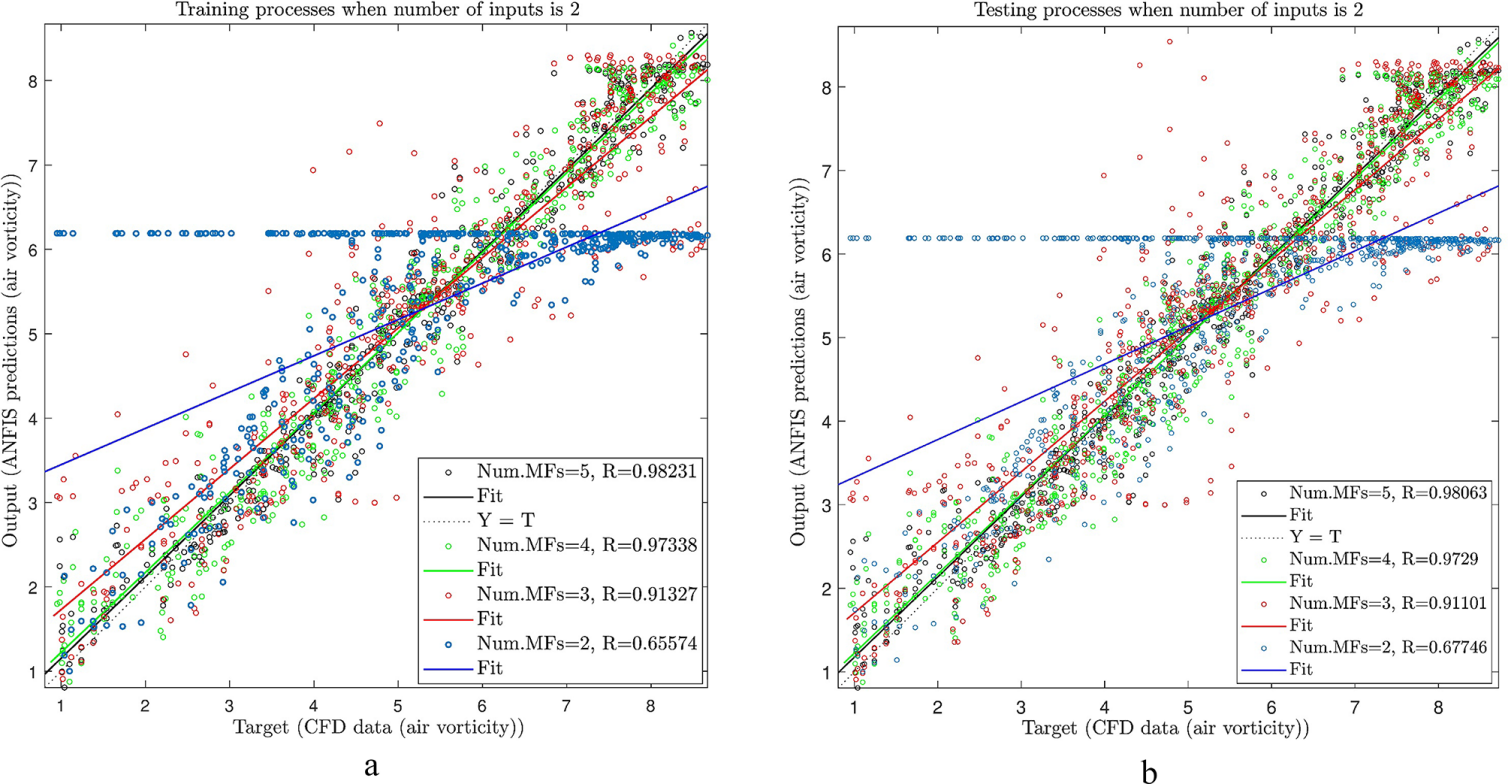
(**a**)Different number of MFs in ANFIS training process (number of inputs = 2, type of MFs is dsigmf) (**b**)Different number of MFs in ANFIS testing process (number of inputs = 2, type of MFs is dsigmf).

**Fig. 8:**
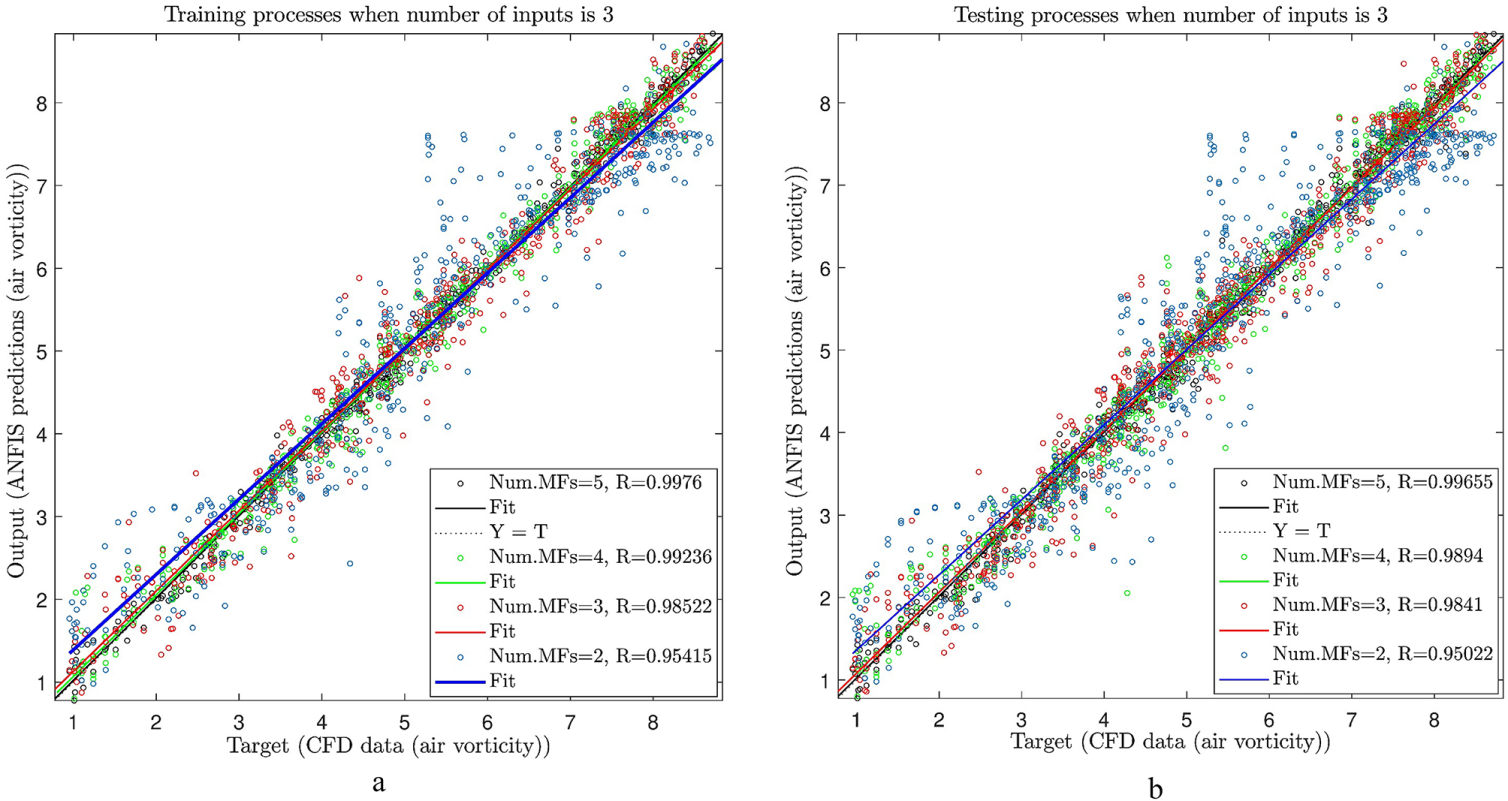
(**a**)Different number of MFs in ANFIS training process (number of inputs = 3, type of MFs is dsigmf) (b) Different number of MFs in ANFIS testing process (number of inputs = 3, type of MFs is dsigmf).

**Fig. 9:**
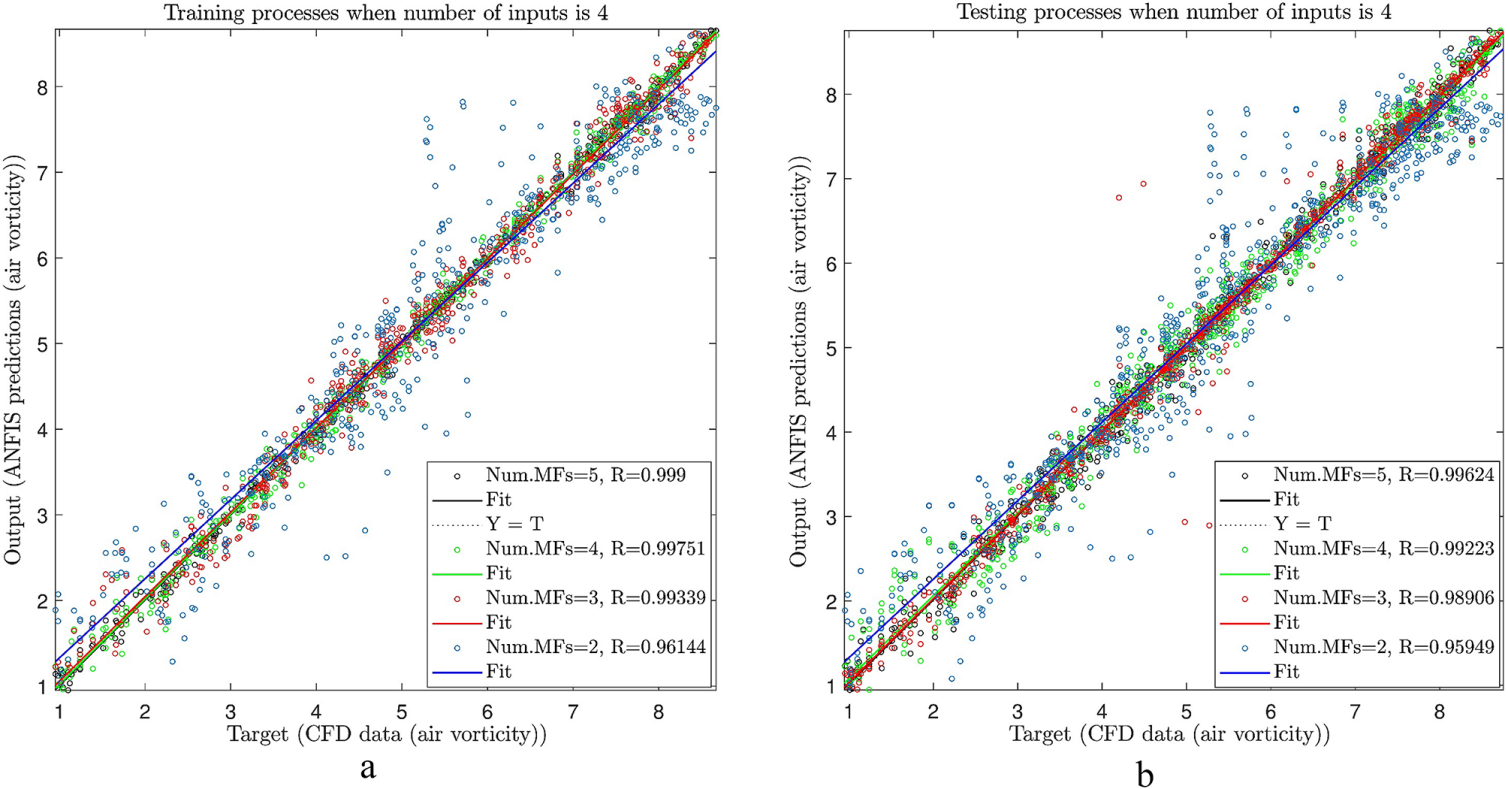
(a)Different number of MFs in ANFIS training process (number of inputs = 4, type of MFs is dsigmf) (**b**) Different number of MFs in ANFIS testing process (number of inputs = 4, type of MFs is dsigmf).

**Fig. 10:**
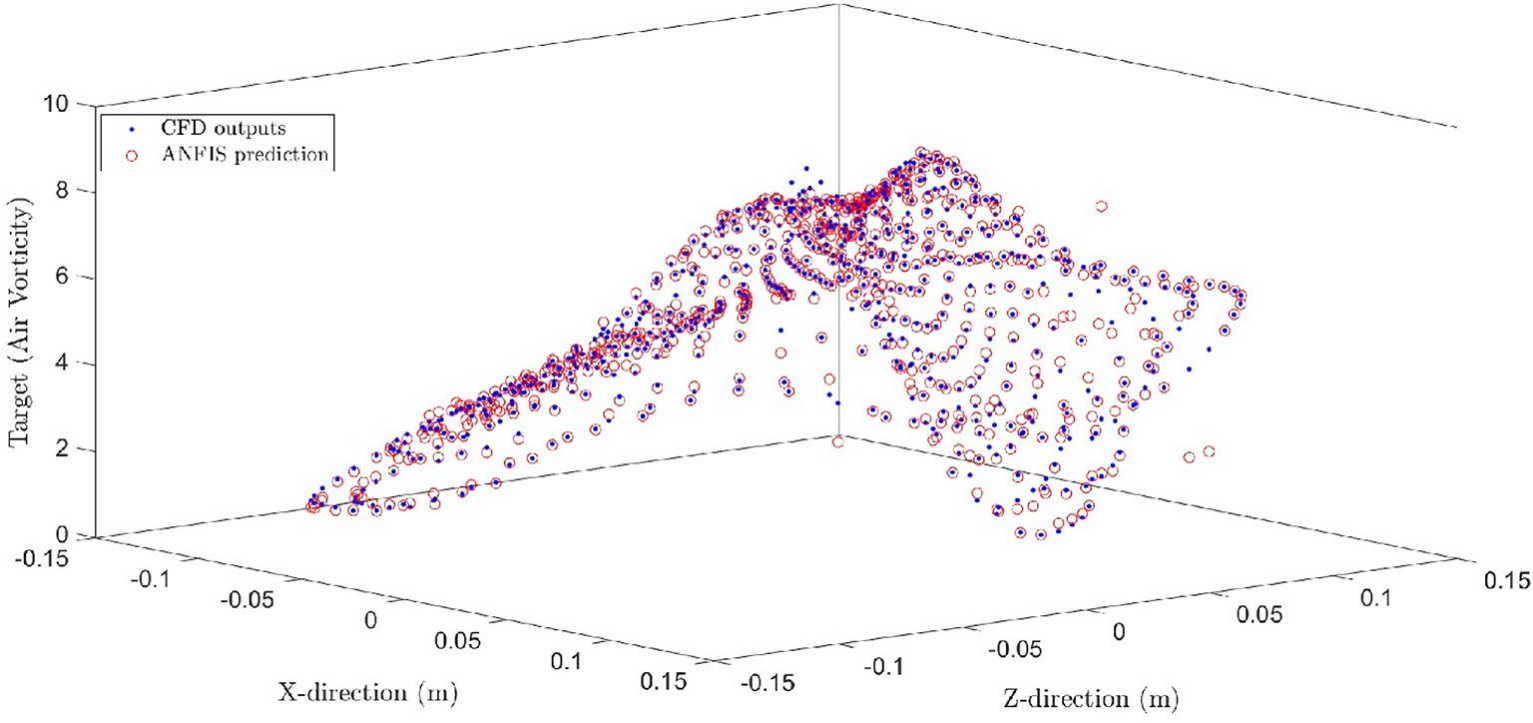
Correlation and comparison of CFD results (ANFIS Target) with ANFIS prediction (output).

**Fig. 11:**
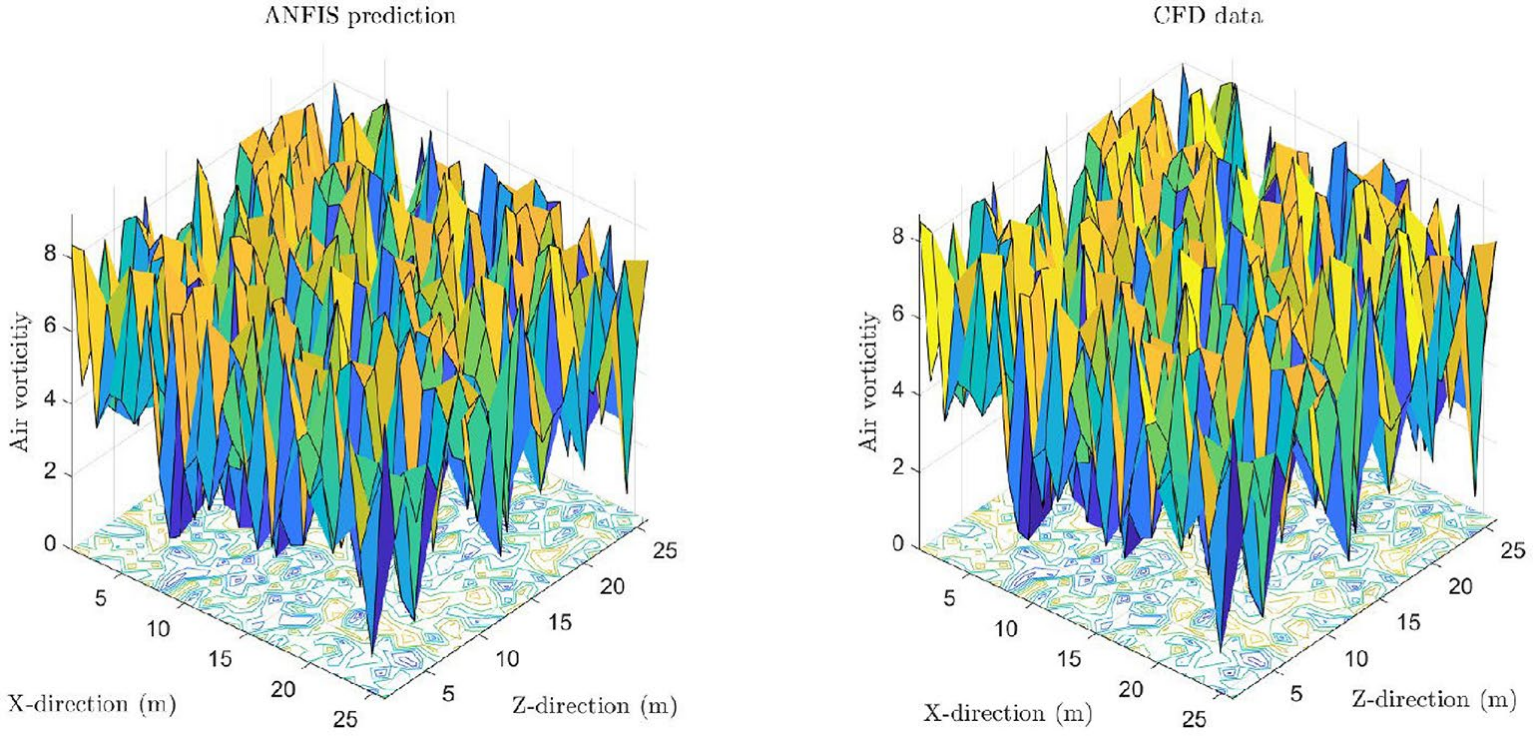
Comparison of ANFIS prediction(left) with CFD results(right) using the best of ANFIS intelligence.

## Data Availability

The datasets used and analysed during the current study are available from the corresponding author upon reasonable request.
